# Distinct Genomic Landscape of Colorectal Mucinous Carcinoma Determined *via* Comprehensive Genomic Profiling: Steps to a New Treatment Strategy

**DOI:** 10.3389/fonc.2021.603564

**Published:** 2021-05-07

**Authors:** Liang Huang, Shuanglin Luo, Xingwei Zhang, Yonghua Cai, Fangqin Xue, Huanxin Hu, Ziwei Zeng, Tengjiao Lin, Fei Wang, Weifeng Wang, Sen Zhang, Liang Kang

**Affiliations:** ^1^ Department of Colorectal Surgery, The Sixth Affiliated Hospital of Sun Yat-Sen University, Guangzhou, China; ^2^ Guangdong Institute of Gastroenterology, The Sixth Affiliated Hospital of Sun Yat-Sen University, Guangzhou, China; ^3^ Guangdong Provincial Key Laboratory of Colorectal and Pelvic Floor Diseases, The Sixth Affiliated Hospital of Sun Yat-Sen University, Guangzhou, China; ^4^ Department of Gastrointestinal Surgery, Fujian Provincial Hospital, Fuzhou, China; ^5^ Department of Research and Development, OrigiMed, Shanghai, China; ^6^ Department of Colorectal Surgery, The First Affiliated Hospital of Guangxi Medical University, Nanning, China

**Keywords:** colorectal cancer, mucinous adenocarcinoma, adenocarcinoma with mucous composition, next-generation sequencing, hypermutated tumor

## Abstract

Colorectal mucinous carcinoma (MC) is associated with inferior prognosis and response to treatment compared to adenocarcinoma (AC). The molecular landscapes of MC and adenocarcinoma with mucous composition (AMC) are not well-defined. We aimed to describe the genomic landscape of MC and AMC in a large colorectal cancer cohort. Tumor samples from patients with MC, AMC, or AC were analyzed using next-generation sequencing. MC had a molecular signature distinct from that of AC; genomic features were similar between AMC and MC but not between AMC and AC. *HER2* amplification and *TP53* and *APC* mutation rates were lower, whereas *SMAD4*, *PIK3CA*, *ACVR2A*, *KMT2D*, *LRP1*, *TGFBR2*, *GRIN2A*, *BRAF* V600E, *PTEN*, and *BRCA2* mutation rates were higher in MC than in AC. The mutation frequencies in MAPK, PI3K, and TGF- pathways were higher, whereas those of cell cycle proteins and Wnt were lower in MC and AMC than in AC. The proportion of hypermutated tumors was significantly higher in MC and AMC than in AC. As MC has a distinct molecular signature from AC, immunotherapy can be potentially applied in treating MC. Similar molecular profiles of AMC and MC suggest that treatment strategies for MC, but not AC, can be used for AMC treatment.

## Introduction

According to the 2018 global cancer statistics released by the International Cancer Research Institute of the World Health Organization (WHO), colorectal cancer (CRC) has the third highest incidence rate and second highest mortality rate, and an increasing annual prevalence rate (1, 2). According to the WHO classification, mucinous carcinoma (MC) is a distinct pathological CRC subtype, with a substantial mucous component of more than 50% of the tumor volume, and accounts for 1015% of all CRC cases (3, 4). MC constitutes a histological subtype with poor differentiation potential and is a predictive factor for poor prognosis (5, 6).

MC is clinically more prevalent among women, frequently located in the proximal colon, and associated with young age, high malignancy grade, tumor infiltration, lymph node metastasis, and peritoneal metastasis (4, 7). Compared with adenocarcinoma not otherwise specified (AC), patients with MC are reportedly less responsive to neoadjuvant radiotherapy and chemotherapy (8). The efficacy of first-line chemotherapy with oxaliplatin or irinotecan is lower among patients with advanced MC than among those with AC. Furthermore, patients with metastatic MC do not benefit from treatment with anti-epidermal growth factor (EGFR) monoclonal antibodies, even in cases with wild-type RAS and BRAF (9). Therefore, it is important to investigate the molecular characteristics of colorectal MC in detail and explore a more effective treatment strategy.

Colorectal MC has unique molecular characteristics. Most early studies focused on protein expression levels and reported that MUC2 and MUC5AC are upregulated in MC tumors (10, 11). Recent genomic analyses have reported that colorectal MC has a higher mutation frequency in Ras/MAPK and PI3K/Akt/mTOR pathways in MC than in AC, with a higher incidence of microsatellite instability (MSI), which is potentially associated with Lynch syndrome and the CpG island methylator phenotype (4). However, owing to limitations in detection technology, previous studies have not revealed the genetic landscape of MC, including comprehensive genomic characteristics, pathway analyses, and biomarkers for immunotherapy. The fraction of mucous composition varies substantially among Colorectal Cancers. Prior studies confirm that the variation of mucous composition in CRC is associated with distinct molecular and clinical features (12, 13). However, adenocarcinomas with relatively low mucous composition (less than 50%, also known as AMC) are usually diagnosed and treated as AC. The somatic mutational landscape of this unique subgroup is less known and the best clinical management of AMC needs to be addressed in the light of the mutational background (4).

In this study, we aimed to perform comprehensive targeted next-generation sequencing (NGS) to detect the two pathological subtypes of CRC, MC and AMC, and gain deep insights into their molecular characteristics, through the evaluation of the landscape of genetic alterations, pathway analysis, and analysis of biomarkers for immunotherapy to provide a molecular basis for the establishment of a precise treatment strategy for MC and AMC.

## Materials and Methods

### Patients and Tumor Selection

Tumor specimens of patients with CRC involved in this study from January 2018 to September 2019 were sent for NGS analysis. Of 2,115 patients with CRC, 1,226 with a confirmed pathological diagnosis of MC, AMC, or AC were selected and recruited. Patients with an uncertain diagnosis of the pathological subtype or those with other special pathological subtypes, such as signet-ring cell carcinoma, undifferentiated carcinoma, and squamous cell carcinoma, were excluded. Of 2,115 patients with CRC, 1,226 with a confirmed pathological diagnosis of MC, AMC, or AC were selected and recruited. Patients with an uncertain diagnosis of the pathological subtype or those with other special pathological subtypes, such as signet-ring cell carcinoma, undifferentiated carcinoma, and squamous cell carcinoma, were excluded. MC was defined as extracellular mucus secretion accounting for >50% of the tumor volume. AMC was defined that accounted for 50%. And AC was defined as tumor with no extracellular mucus secretion. All tumor tissues were assessed independently by two experienced pathologists before sample disposal to pathologically confirm the diagnoses.

This study was approved by the Institution Review Board of the Sixth Affiliated Hospital of Sun Yat-sen University in accordance with the Declaration of Helsinki. Written informed consent was obtained from all enrolled patients.

### NGS Analysis

NGS analysis was carried out at OrigiMed (Shanghai, China), a College of American Pathologists-accredited and Clinical Laboratory Improvement Amendments-certified laboratory, using a 450-gene comprehensive assay (14). At least 50 ng of DNA was extracted from each 40mm formalin-fixed paraffin-embedded (FFPE) tumor sample using a DNA Extraction Kit (QIAamp DNA FFPE Tissue Kit) in accordance with the manufacturers protocols. This panel encompassed all coding exons of 450 cancer-related genes and 64 selected introns of 39 genes that are frequently rearranged in solid tumors. Furthermore, the probe density was increased to ensure high capture efficiency in the conservatively low-read-depth regions. Peripheral blood was sampled from each patient as the normal control sample for genomic profiling. The genes were captured and sequenced with a mean coverage of 900 for FFPE samples and 300 for matched blood samples using an Illumina NextSeq 500 Platform (Illumina Incorporated, San Diego, CA, USA).

### Genetic Analysis

All types of genetic alterations, including single-nucleotide variant (SNV), short and long indels, copy number alterations (CNAs), and gene rearrangement, were called using a suite of bioinformatics pipelines. Analysis of SNVs and indels began with the alignment of raw reads to the human genome reference sequence (hg19) with the Burrows-Wheeler Aligner (v0.62; BWA, Cambridge, MA, USA), followed by polymerase chain reaction (PCR) duplicates removal using MarkDuplicates algorithm from Picard (version 1.47; Cambridge, MA, USA). Local realignment and base quality recalibration for SNVs were performed using GATK (v3.1-1; Cambridge, MA, USA) and subsequently called by MUTECT (v1.7; Cambridge, MA, USA). The CNAs included: (1) amplification, defined as an increase in the number of gene segment copies by 8, and (2) homozygous deletion, defined as decrease of complete loss of gene segment copies in samples with 20% purity. To identify these alterations, tumor cellularity was estimated by allele frequencies of sequenced single-nucleotide polymorphisms (SNPs). For detection of gene rearrangement, aligned reads with abnormal insert size of 2,000 or zero bp were collected and used as discordant reads, that is, paired-end reads that could not be closely mapped to a genome reference, with each read of paired reads aligned to the same chromosomes or different chromosomes. Originally, the discordant reads with the distance less than 500 bp formed clusters were further assembled by fermi-lite to identify potential rearrangement breakpoints. The breakpoints were double confirmed by BLAT, and the resulting chimeric gene candidates were annotated. For germline mutations, common single nucleotide polymorphisms, defined as those from the dbSNP database (Version 147), at a frequency of more than 1.5% from the Exome Sequencing Project 6500 (ESP6500), or at a frequency of more than 1.5% from the 1000 Genomes Project, were excluded. Furthermore, the variant allele frequency was adjusted with tumor purity estimated using FACETS.

### Tumor Mutational Burden (TMB) and MSI

The TMB was estimated using the method of Chalmers etal. (15). In brief, the somatic, coding, base substitution, and short indel mutations were enumerated. Driver mutations and germline alterations in the dbSNP database were not enumerated. The TMB was determined by dividing the total number of mutations by the size of the coding region. The MSI status was determined in all cases. Based on the MSI score, samples were classified as MSI-high (MSI-H) and microsatellite stable (MSS).

### Statistical Analyses

Qualitative variables were assessed using Fishers exact test. Normally distributed quantitative data were analyzed using the *t*-test and non-normally distributed data were analyzed using the Wilcoxon rank test. All tests were two-tailed and significance was defined as a *P* value less than 0.05. All statistical analyses were performed using R software (Version 3.4.2).

## Results

### Clinical Characteristics

We defined MC as adenocarcinoma with mucous composition greater than 50% and AC as adenocarcinoma with no mucous composition. Adenocarcinoma with mucous composition but less than 50% is called AMC. **Table 1** summarizes the characteristics of the patients. In total, 1,226 patients with CRC were enrolled in the study and divided into three categories by histological subtype: MC (10.5%), and AMC (8.2%), and AC (81.3%). The median age of patients with MC was less than that of patients with AC (56 *vs.* 59 years, *P* = 0.037), and the incidence of MC in the right colon was higher than that of AC (41.9 *vs.* 24.2%, *P* < 0.001). Patients with MC accounted for a larger proportion of patients with stage III CRC (44.2%) than AC (29.9%, *P* < 0.001) and AMC (29.0%, *P* = 0.091); but for AMC, the difference is only marginally significant. Furthermore, AMC was significantly more common in the right colon than AC was (50 *vs.* 24.2%, *P* < 0.001). No significant differences were observed between AMC and AC with respect to other clinical features.

**Table 1:** Patient and tumor characteristics.

Characteristics	MCN = 129 (%)	AMCN = 100 (%)	ACN = 997 (%)	*P* value MC *vs.* AC	*P* value AMC *vs.* AC	*P* value MC *vs.* AMC
**Gender**				1.000	0.134	0.227
**Female**	50 (38.8)	47(47)	390 (39.1)			
**Male**	79 (61.2)	53(53)	607 (60.9)			
**Age**				0.037	0.399	0.043
**Median**	56	62	59			
**Range**	1786	2882	1696			
**Primary Tumor Site**				<0.001	<0.001	0.016
**Left colon**	55 (42.6)	25 (25)	320 (32.1)			
**Right colon**	54 (41.9)	50 (50)	241 (24.2)			
**Rectum**	20 (15.5)	24 (24)	427 (42.8)			
**NA**	0 (0)	1 (1)	9 (0.9)			
**Stage at diagnosis** **^a^**				<0.001	0.133	0.091
**Stage I**	3 (2.3)	5 (5)	58 (5.8)			
**Stage II**	40 (31)	33 (33)	232 (23.3)			
**Stage III**	57 (44.2)	29 (29)	298 (29.9)			
**Stage IV**	27 (20.9)	32 (32)	367 (36.8)			
**NA**	2 (1.6)	1 (1)	42 (4.2)			
**Sample Source**				0.415	0.622	0.177
**Primary lesion**	126 (97.6)	100 (100)	984 (98.7)			
**Metastatic lesion**	3 (2.4)	0 (0)	13 (1.3)			

a Stage at diagnosis based on AJCC (8th edition). MC, mucinous carcinoma; AMC, adenocarcinoma with mucous composition; AC, adenocarcinoma; AJCC, American Joint Committee on Cancer; NA, not applicable.

### Comparison of Common Gene Mutations Among MC, AMC, and AC

**Figure 1:**
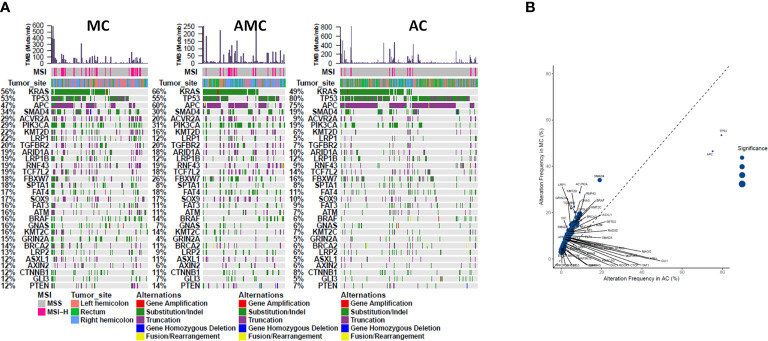
Comparison of common gene mutations in MC, AMC, and AC. **(A)** Genomic landscape showing mutated genes among MC, AMC, and AC. Each column denotes an individual tumor and each row represents the MSI status, tumor site, and individual genes. Colors indicate the type of genetic alterations as indicated in the legend. **(B)** Analysis of the gene alteration frequency showing a higher gene alteration frequency in the MC group. MC, mucinous carcinoma; AMC, adenocarcinoma with mucous composition; AC, adenocarcinoma; MSI, microsatellite instability.

Comprehensive targeted NGS revealed that the top 10 prevalent mutations in MC were *KRAS* (55.8%), *TP53* (53.5%), *APC* (46.5%), *SMAD4* (34.1%), *ACVR2A* (28.7%), *PIK3CA* (28.7%), *KMT2D* (22.5%), *LRP1* (21.7%), *TGFBR2* (20.2%), and *ARID1A* (19.4%) (**Figure 1A**). In general, the mutation profile of MC was different from that of AC (**Figure 1B**); however, the mutation profiles of MC and AMC did not differ significantly (data not shown). Among the commonly mutated genes in CRC, *TP53* (53.5 *vs.* 79.5%, *P* < 0.001) and *APC* (46.5 *vs.* 75.1%, *P* < 0.001) displayed a significantly lower mutation rate, whereas *SMAD4* (34.1 *vs.* 19.1%, *P* < 0.001), *PIK3CA* (28.7 *vs.* 19.2% *P* = 0.014), *ACVR2A* (28.7 *vs.* 9.1%, *P* < 0.001), *KMT2D* (22.5 *vs.* 6.4%, *P* < 0.001), *LRP1* (21.7 *vs.* 4.8%, *P* < 0.001), *TGFBR2* (20.2 *vs.* 6.4%, *P* < 0.001), and *GRIN2A* (14.7 *vs.* 4.5%, *P* < 0.001) displayed a significantly higher mutation rate in MC than in AC.

**Figure 2:**
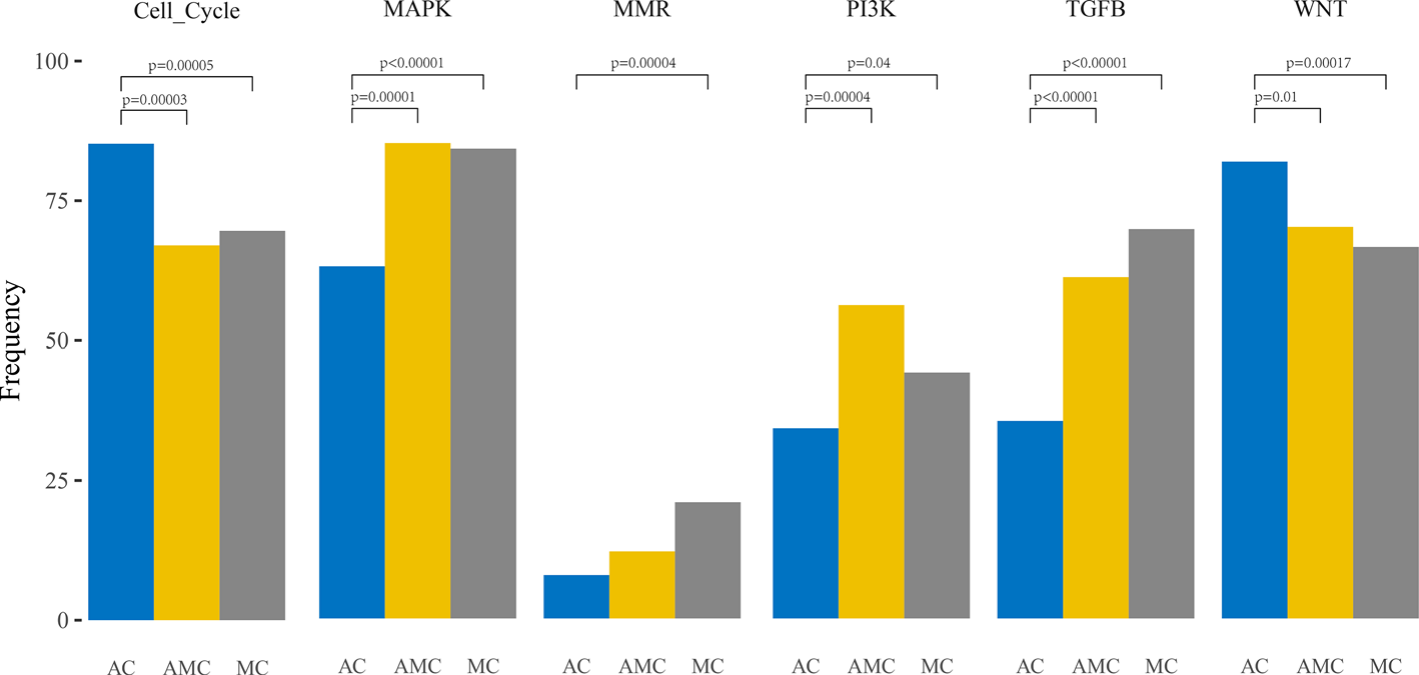
Comparative analysis of the frequencies of genetic alterations based on signaling pathways playing an important role in oncogenesis in colorectal cancer.

Furthermore, pathway analysis revealed that the mutation frequencies in MAPK, PI3K, and TGF pathways were higher, whereas those of cell cycle proteins and the Wnt pathway were lower, in MC and AMC than in AC (**Figure 2**).

### Comparison of Clinically Actionable Alterations Among MC, AMC, and AC

The mutation pattern of clinically actionable alterations in MC was different from that in AC but similar to that in AMC. The mutation rates of *BRAF* V600E (10.9 *vs.* 3.3%, *P* < 0.001), *PIK3CA* (28.7 *vs.* 19.2%, *P* = 0.014), *PTEN* (14.7 *vs.* 7.2%, *P* = 0.027), and *BRCA2* (17.8 *vs.* 5.5%, *P* < 0.001) were significantly higher in MC than in AC. Although *HER2* mutation rates were comparable between MC and AC (3.9 *vs.* 6.2%, *P* = 0.423), *HER2* amplification occurred at a rate of 2.1% in AC but was not detected in MC or AMC. The mutation rate of KRAS was significantly higher in AMC than in AC (65.0 *vs.* 49.2%, *P* = 0.001); however, it did not significantly differ between MC and AC or MC and AMC. The mutation frequencies of clinically actionable genes in MC, AMC, and AC are summarized in **Table 2**.

**Table 2:** Comparison of clinically actionable gene alterations in MC, AMC, and AC.

Genes	MCN = 129 (%)	AMCN = 100 (%)	ACN = 997 (%)	P value MC *vs.* AC	P value AMC *vs.* AC	P value MC *vs.* AMC
***KRAS***	72 (55.8%)	65 (65.0%)	491 (49.2%)	0.135	0.001	0.135
***NRAS***	4 (3.1%)	2 (2.0%)	36 (3.6%)	1.000	0.570	0.698
***VEGFA***	2(1.6%)	3(3.0%)	13(1.3%)	0.687	0.173	0.656
***EGFR***	9(7.0%)	2(2.0%)	100 (10.0%)	0.168	0.231	0.072
***BRAF*** **V600E**	14 (10.9%)	8 (8.0%)	33 (3.3%)	<0.001	0.027	0.507
***BRAF*** **non-V600E**	6 (4.7%)	5 (5.0%)	22 (2.2%)	0.123	0.091	1.000
***HER2 (ERBB2)*** **amplification**	0 (0.0%)	0 (0.0%)	21 (2.1%)	0.158	0.248	NA
***HER2 (ERBB2)*** **mutation**	5 (3.9%)	4 (4%)	62 (6.2%)	0.423	0.508	1.000
**All druggable** **fusion**	2 (1.6%)	1 (1%)	17 (1.7%)	1.000	1.000	1.000
***NTRK1*** **fusion**	0 (0%)	0 (0%)	5 (0.5%)	0.039	0.689	NA
***NTRK3*** **fusion**	1 (0.8%)	0 (0%)	1 (0.1%)	0.036	1.000	0.506
***PIK3CA***	37 (28.7%)	31 (31.0%)	191 (19.2%)	0.014	0.008	0.771
***AKT1***	5 (3.9%)	3 (3%)	22 (2.2%)	0.224	0.491	1.000
***PTEN***	19 (14.7%)	14 (14%)	72 (7.2%)	0.027	0.013	0.844
***BRCA1***	6 (4.7%)	5 (5%)	19 (1.9%)	0.057	0.06	1.000
***BRCA2***	23 (17.8%)	9 (9%)	55 (5.5%)	<0.001	0.043	0.553

MC, mucinous carcinoma; AMC, adenocarcinoma with mucous composition; AC, adenocarcinoma; NA, not applicable.

Gene fusions in receptor tyrosine kinases have been recently identified as druggable targets in CRC (16). One patient with an MC tumor in the right colon harbored an *ETV6-NTRK3* fusion and the tumor was identified as MSI-H. *NCOA4-RET* and *FGFR2-PIBF1* fusions were observed in patients with MC and AMC, respectively. The frequency of druggable fusions did not significantly differ among the three CRC pathological subtypes.

### Comparison of Immune Biomarkers in MC, AMC, and AC

We defined hypermutated tumors as MSI-H tumors or those harboring *POLE* mutations that result in a dramatic TMB elevation. In general, the proportion of hypermutated tumors was significantly higher in MC and AMC than in AC (MC 27.9% *vs.* AC 8.4%, *P* < 0.001; AMC 18% *vs.* AC 8.4%, *P* = 0.003).

**Figure 3:**
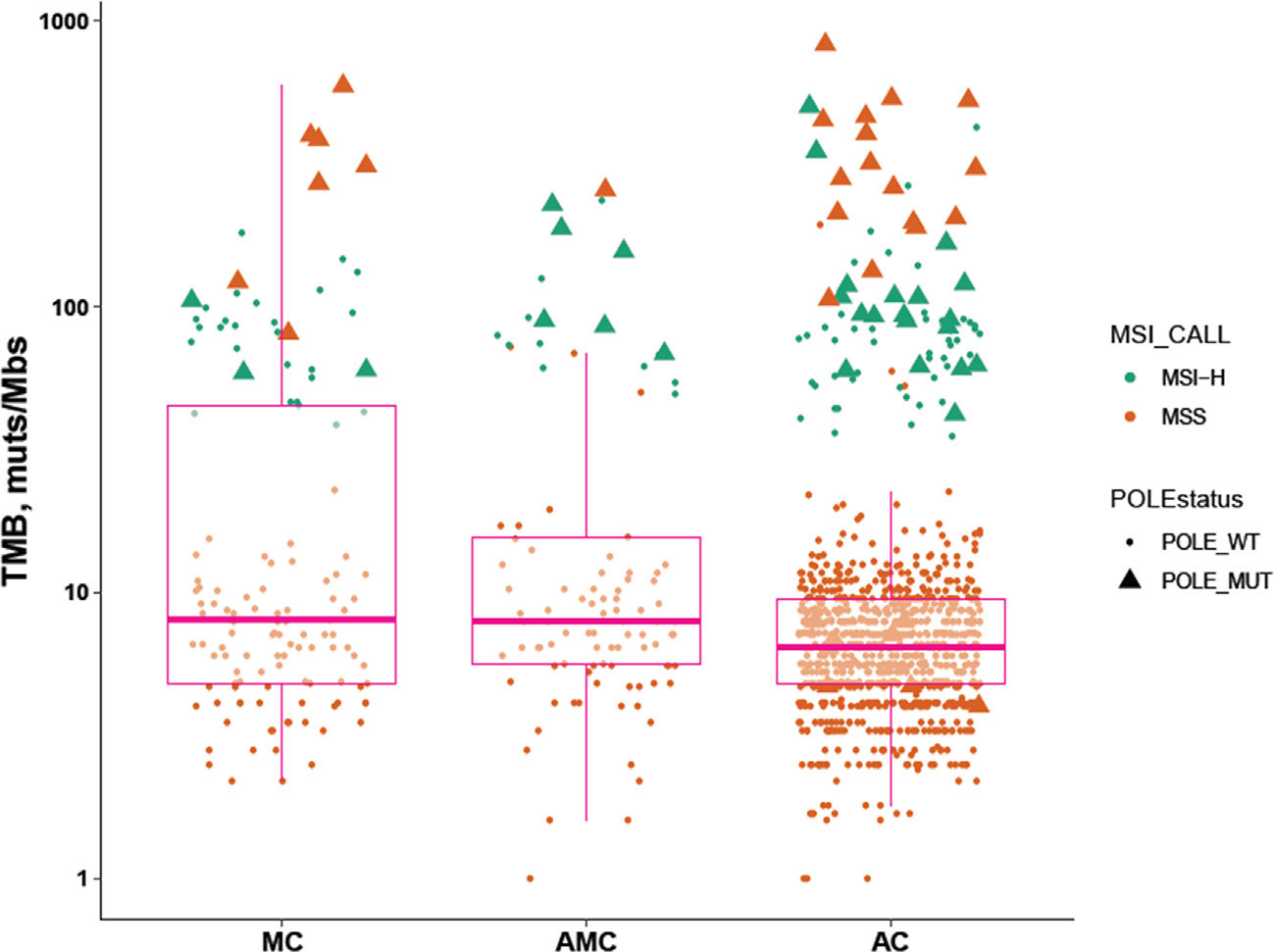
Comparison of immunotherapy-related biomarkers in MC, AMC, and AC. MC, mucinous carcinoma; AMC, adenocarcinoma with mucous composition; AC, adenocarcinoma; MSI, microsatellite instability; TMB, tumor mutational burden; MSS, microsatellite stability.

The percentage of MSI-H tumors was significantly higher in MC and AMC than in AC (MC 22.5% *vs.* AC 6.8%, *P* < 0.001; AMC 17% *vs.* AC 6.8%, *P* = 0.001) and comparable between MC and AMC. Although the percentage of all *POLE* mutations among the three subtypes did not differ significantly, the proportion of *POLE* mutations resulting in a high TMB in MSS tumors was significantly higher in MC than in AC (5.4 *vs.* 1.6%, *P* = 0.004). The median TMB and median number of somatic mutations were also significantly higher in MC and AMC than in AC (**Figure 3** and **Table 3**).

**Table 3:** Comparison of immunotherapy-related biomarkers in MC, AMC, and AC.

Characteristics	MCN = 129 (%)	AMCN = 100 (%)	ACN = 997 (%)	P value MC *vs.* AC	P value AMC *vs.* AC	P value MC *vs.* AMC
**Hypermutated tumor** ^a^	36 (27.9)	18 (18.0)	84 (8.4)	<0.001	0.003	0.086
**MSI-H tumor**	29 (22.5)	17 (17.0)	68 (6.8)	<0.001	0.001	0.324
***POLE*** **ALL mutation**	10 (7.8)	7 (7.0)	43 (4.3)	0.117	0.209	1.000
***POLE*** **Hypermutation in MSS tumor** ^b^	7 (5.4)	1 (1)	16 (1.6)	0.004	1.000	0.074
**TMB**				<0.001	<0.001	0.967
**Median (muts/Mb)**	7.0	6.9	5.4			
**Range**	1.2591.5	0254.7	0825.3			
**Somatic mutations number**				0.002	0.003	0.963
**Median (N/tumor)**	9	9	8			
**Range**	2277	1160	1269			

a Hypermutated tumors are defined as MSI-H tumors or tumors harboring POLE mutations, resulting in drastic TMB elevation.

b Hypermutation in MSS tumors associated with POLE-mutated cases with dramatic TMB elevation in MSS CRC, mostly caused by POLE mutations in the exonuclease domain.

MC, mucinous carcinoma; AMC, adenocarcinoma with mucous composition; AC, adenocarcinoma; CRC, colorectal cancer; MSI, microsatellite instability; TMB, tumor mutational burden; MSS, microsatellite stability.

### Hypermutated Tumors in MC

**Figure 4:**
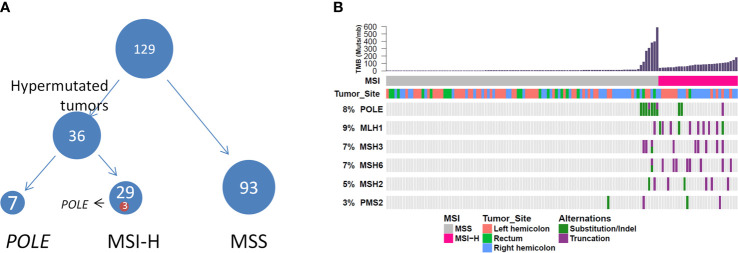
**(A)** Hypermutated tumors include MSI-H tumors and tumors harboring *POLE* mutations. **(B)** Genomic landscape showing associations among MSI-H, microsatellite instability, high TMB, MSI status, and *POLE* mutation. TMB, tumor mutational burden.

We further evaluated the relevant immunotherapy indicators in MC, which revealed that 29 of 129 patients harbored MSI-H tumors, among which three harbored *POLE* mutations. Seven of 100 patients harbored MSS tumors with *POLE* mutations (**Figure 4**). Moreover, all *POLE* mutations detected in MSS tumors were located in the exonuclease domain, which led to extremely high levels of TMB. Only one E972G mutation in an MSS tumor was not located in the exonuclease domain and the TMB in this case was relatively lower (79.5 muts/Mb) than that in cases of *POLE* mutations in the exonuclease domain (TMB range, 121.1595.5 muts/Mb). Furthermore, *POLE* mutations detected in three patients harboring MSI-H tumors were not present in the exonuclease domain. Details of clinical and molecular characteristics of MC with *POLE* mutations are summarized in **Table 4**.

**Table 4:** Clinical and molecular characteristics of MC with *POLE* mutations.

Number	Gene	Sex	Age(years)	Primary tumor site	Stage	TMB(muts/Mb)	MSI	Variation type	DNA change	Amino acid change
**1**	*POLE*	female	51	Right colon	I	57.7	MSI-H	Substitution	c.5648C>T	p.A1883V
**2**	*POLE*	male	43	Right colon	III	58.9	MSI-H	Substitution	c.557C>T	p.A186V
**3**	*POLE*	female	40	Left colon	II	104	MSI-H	Truncation	c.4337_4338del	P.V1446Gfs*3
**4**	*POLE*	female	37	Rectum	III	396	MSS	Substitution	c.1231G>T	p.V411L
**5**	*POLE*	female	59	Left colon	II	268.6	MSS	Substitution	c.857C>G	p.P286R
**6**	*POLE*	female	41	Rectum	III	121.1	MSS	Substitution	c.1231G>C	p.V411L
**7**	*POLE*	female	56	Right colon	II	79.5	MSS	Substitution	c.2915A>G	p.E972G
**8**	*POLE*	male	47	Right colon	II	383.4	MSS	Substitution	c.1231G>T	p.V411L
**9**	*POLE*	female	47	Right colon	II	591.5	MSS	Substitution	c.857C>G	p.P286R
**10**	*POLE*	male	76	Left colon	I	309.5	MSS	Substitution	c.857C>G	p.P286R

MC, mucinous carcinoma; TMB, tumor mutational burden; MSI, microsatellite instability.

## Discussion

This study identified the comprehensive genomic features of MC and AMC by targeted NGS using a large cohort of patients with CRC. We comprehensively compared genetic differences between MC, AMC, and AC and identified the following major features. In general, MC had a molecular signature that was distinct from that of AC. The genomic features were similar between AMC and MC but different between AMC and AC. MC had a distinguished mutation pattern for prevalent gene mutations and biomarkers used clinically for CRC. Most importantly, the proportion of hypermutated tumors in MC and AMC was significantly higher than that in AC, indicating the higher applicability of immunotherapy for patients with these histological subtypes. Our results support developing more tailored treatment strategies for patients with CRC according to an individuals histological subtype.

Previous studies have suggested that mutations in *SMAD4*, *GNAS*, *BRAF*, and *KRAS* occur at high frequencies in MC, whereas *TP53*, *APC*, and *NRAS* mutations are less common (17, 18). The high frequency of *BRAF* mutations in MC is well-documented in the literature and supported by our findings (19, 20). Patients with metastatic CRC harboring a *BRAF* V600E mutation have a significantly worse prognosis. This study found the *BRAF* V600E mutation rate was significantly higher in MC and AMC than in AC, whereas the mutation rate of *BRAF* (non-V600E) did not significantly differ among the three groups. The *SMAD4* mutation frequency was significantly higher in MC and AMC than in AC. Patients with a *SMAD4* deletion have worse relapse-free survival and are resistant to chemotherapy with 5-fluorouracil (21). MCs were associated with an unsatisfactory response to neoadjuvant chemotherapy. However, whether *SMAD4* plays a role in chemotherapy resistance mechanisms needs further research. On the other hand, the stage of the cancer is significantly associated with the frequency of specific mutation. For example, *BRAF* (V600E) is more frequent in high-stage MC. The observation suggests that the variation in the mutation rates among the three cancer types is attributed to the different clonal evolution processes, from which MC arises as a unique subtype.

A recent study reported that approximately 5% of patients with CRC harbor a *HER2* mutation. In patients with CRC, nearly half of *HER2* alterations are mutation rather than amplification or protein overexpression. Herein, *HER2* amplification was not observed in MC. However, a proportion of patients with MC harbored *HER2* mutations. Previous animal experiments have reported that the growth of implanted tumors harboring mutant *HER2* can be inhibited by HER2 inhibitors, including trastuzumab, lapatinib, and afatinib, alone and in combination with trastuzumab and tyrosine kinase inhibitors (22,24).

Immune checkpoint inhibitors (ICIs) have recently been widely used in solid and hematological malignancies (25). We defined hypermutated tumors as MSI-H tumors or those harboring *POLE* mutations that result in a dramatic TMB elevation, as there is robust evidence for MSI-H and *POLE* mutations as predictive biomarkers for a good response to immunotherapy in CRC (26, 27). Pembrolizumab has been approved for treating solid tumors with MSI-H/deficient mismatch repair (dMMR) and nivolumab ipilimumab has been approved for treating advanced CRC with MSI-H/dMMR (28, 29). Recently, a study on neoadjuvant treatment of CRC was conducted using a combinatorial treatment with an anti-PD-1 antibody and anti-CTLA-4 antibody. The treatment resulted in a pathological response in 20/20 patients and primary pathological remission in 19/20 patients with dMMR tumors (30). MC is significantly more likely to be associated with MSI-H in the colon and rectum (20). The proportion of MSI-H tumors in this study was significantly higher in MC and AMC than in AC, suggesting that immunotherapy is suitable for a larger proportion of patients with MC and AMC. MC was more prevalent in stage III CRC in this study, indicating that patients are more likely to develop local lymph node metastasis and present locally advanced CRC. In some cases of locally advanced CRC, it is challenging for surgeons to perform R0 (margin-negative) resection, which results in a worse prognosis for patients. ICIs in a neoadjuvant setting would be an effective treatment alternative for patients with MC with MSI-H/dMMR; thus, it is necessary to clarify the MSI/MMR status before any treatment.

Immunotherapy in MSS CRC tumors still lacks efficacy; therefore, there is an urgent need to identify biomarkers for immunotherapy in MSS tumors. Hypermutation in MSS CRCs is often associated with *POLE* mutations accompanied by dramatic TMB elevation, owing to the loss of DNA replication fidelity caused by *POLE* mutations in the exonuclease domain (27). Wang etal. summarized the *POLE/POLD1* mutation rate in 47,721 patients with different cancer types and identified that patients harboring *POLE/POLD1* mutations have a significantly higher TMB. When adjusting for cancer types and MSI status for multivariate Cox regression analysis, *POLE/POLD1* mutations were found to be independent risk factors for identifying patients that could benefit from ICI treatment (27). In this study, the frequency of *POLE* mutations resulting in high TMB in MSS tumors was significantly higher in MC than in AC. In addition, the proportion of hypermutated tumors (MSI-H or *POLE* mutations) was 27.9% in MC, suggesting that up to 30% of patients with CRC MC may benefit from immunotherapy. Furthermore, the mutation pattern of *POLE* differed between MSS and MSI-H tumors and *POLE* mutations occurring in the exonuclease domain markedly increased the TMB in MSS tumors.

We acknowledge that the current study had several limitations. First, the retrospective study design could not exclude selection bias. Second, the clinical data of patients treatments and outcomes were not controlled and collected in the current study; therefore, the clinical impacts of our findings need further confirmation. Finally, on the potential benefit of ICI, the effects of tumor infiltrating lymphocytes need to be evaluated along with the mutational profile as well as MSI and POLE statuses; whereas in the current study still lacks the pathological data for tumor immune microenvironment. On the other hand, the somatic mutational landscape is also affected by the host immune environment, hence serve as a proxy to the activities of the immune cells.

In spite of the limitations, using the large cohorts of MC (*n* = 129) and AMC (*n* = 100) *via* a comprehensive targeted NGS panel, our results reveal the molecular landscapes of MC, AMC, and AC, which could lead to tailored treatment for different histological subtypes of CRC. The selection of baseline clinical and pathological characteristics was relatively intact in this study, allowing the analysis of clinical and genomic features. Our findings shed new light on the treatment and management of patients with MC and AMC. Further prospective studies in patients with MC and AMC are warranted to validate our findings, especially regarding the potential use of immunotherapy.

## Conclusions

We identified a distinct genomic landscape in colorectal MC *via* comprehensive genomic profiling for commonly mutated and clinically actionable genes. Hypermutated tumors account for nearly 30% of MC, suggesting that a large proportion of patients with MC may benefit from immunotherapy; therefore, there is a need for comprehensive molecular testing in these patients. AMC has similar genomic features to MC but different from AC, suggesting the potential for the use of MC treatment strategies for treating AMC.

## Data Availability Statement

The data presented in the study are deposited in the CNGB Sequence Archive, repository, accession number is CNP0001753. The reviewer link is http://db.cngb.org/cnsa/review/show/CNP0001753_20210422_a35ddad7.

## Ethics Statement

The studies involving human participants were reviewed and approved by Institution Review Board of the Sixth Affiliated Hospital of Sun Yat-Sen University. The patients/participants provided their written informed consent to participate in this study.

## Author Contributions

Study design: LH, LK, SZ, and TL. Study recruitment, clinical sample, and data acquisition: XZ, YC, FX, HH, ZZ, SZ, and TL. Bioinformatic analysis: WW and FW. Primary results interpretation: LH and SL. Manuscript drafting: SL, LH, LK, and SZ. All authors contributed to the article and approved the submitted version.

## Funding

This study was supported by the Fundamental Research Funds for the Central Universities, grant number 16ykjc25, Sun Yat-Sen University Clinical Research 5010 Program, grant number 2016005 and Province Natural Science Fund of Guangdong (2018A030313621).

## Conflict of Interest

TL, FW and WW were employed by OrigiMed.

The remaining authors declare that the research was conducted in the absence of any commercial or financial relationships that could be construed as a potential conflict of interest.
